# Factors associated with successful home discharge after inpatient rehabilitation in frail older stroke patients

**DOI:** 10.1186/s12877-020-1422-6

**Published:** 2020-01-23

**Authors:** Tom P. M. M. Vluggen, Jolanda C. M. van Haastregt, Frans E. S. Tan, Gertrudis I. J. M. Kempen, Jos M. G. A. Schols, Jeanine A. Verbunt

**Affiliations:** 1 Department of Health Services Research, Maastricht University, PO Box 616 6200, MD Maastricht, The Netherlands; 2 Care and Public Health Research Institute (CAPHRI), Maastricht University, Maastricht, The Netherlands; 3 Department of Methodology and Statistics, Maastricht University, Maastricht, The Netherlands; 4 Adelante, Centre of Expertise in Rehabilitation and Audiology, Hoensbroek, The Netherlands; 5 Department of Rehabilitation Medicine, Maastricht University, Maastricht, The Netherlands

**Keywords:** Stroke, Rehabilitation, Older people, Prediction, Discharge destination, Community

## Abstract

**Background:**

Stroke is a highly prevalent disease among older people and can have a major impact on daily functioning and quality of life. When community-dwelling older people are hospitalized due to stroke, discharge to an intermediate care facility for geriatric rehabilitation is indicated when return to the previous living situation is expected but not yet possible. However, a substantial proportion is still unable to return home after discharge and has to be admitted to a residential care setting. This study aims to identify which factors are associated with home discharge after inpatient rehabilitation among frail and multimorbid older stroke patients.

**Methods:**

This study is a longitudinal cohort study among 92 community-dwelling stroke patients aged 65 years or over. All patients were admitted to one of eight participating intermediate care facilities for geriatric rehabilitation, under the expectation to return home after rehabilitation. We examined whether 16 potentially relevant factors (age; sex; household situation before admission; stroke history; cardiovascular disorders; diabetes mellitus; multimorbidity; cognitive disability; neglect; apraxia; dysphagia; urinary and bowel incontinence; emotional problems; sitting balance; daily activity level; and independence in activities of daily living) measured at admission were associated with discharge to the former living situation. Logistic regression analysis was used for statistical analysis.

**Results:**

Mean age of the patients was 79.0 years (SD 6.4) and 51.1% was female. A total of 71 patients (77.1%) were discharged to the former living situation within 6 months after the start of geriatric rehabilitation. Of the 16 factors analysed, only a higher level of independence in activities of daily living at admission was significantly associated with home discharge.

**Conclusions:**

Our study shows that the vast majority of previously identified factors predicting home discharge among stroke patients, could not predict home discharge among a group of frail and multimorbid older persons admitted to geriatric rehabilitation. Only a higher level of independence in activities of daily living at admission was significantly related to home discharge. Additional insight in other factors that might predict home discharge after geriatric rehabilitation among this specific group of frail older stroke patients, is needed. Trial registration: ISRCTN ISRCTN62286281. Registered 19-3-2010.

## Background

Stroke is a highly prevalent disease among older people and can have a major impact on daily functioning and quality of life. The prevalence of stroke among Dutch people of 65 years or older is estimated at 54 per 1000 males and 40 per 1000 females [1]. In the Netherlands, after admission to a hospital, about one third of older stroke patients is referred to an intermediate care facility for (geriatric) rehabilitation, which is specifically aimed at the rehabilitation of frail and multimorbid community-dwelling older people [2].

In the Netherlands, admission to an intermediate care facility for geriatric rehabilitation is indicated for community-dwelling frail older people, who are expected to have the capacity to improve to a functional level that enables discharge to their former living situation within a maximum of 6 months of rehabilitation [2]. However, adequately predicting functional recovery and home discharge for this group of older people is a challenge for care professionals, due to the multimorbidity and frailty of these patients. As a result, ultimately up to 25% of these older stroke patients appears not to be able to return to their previous living situation after geriatric rehabilitation [3]. Often, these patients are admitted to a nursing home or other residential care setting [4, 5]. More insight into factors associated with home discharge of frail and multimorbid older stroke patients after geriatric rehabilitation is needed to support care professionals to make an adequate prognosis of discharge destination and to support them to focus their treatment on increasing the chances of home discharge.

Although various studies have assessed predictors of discharge destination of stroke patients, the number of studies conducted exclusively in frail and multimorbid stroke patients in geriatric rehabilitation is limited compared to the much larger body of literature performed among the general population of stroke patients.

However, studies among such frail and multimorbid older patients admitted to intermediate care facilities for rehabilitation, show that the following factors are negatively associated with home discharge; high age [5, 6], female sex [7], living alone [7–10], absence of social support [7, 9–11], hemorrhagic stroke [7], loss of conciousness [8], cognitive disability [6–10, 12], neglect [5, 7, 8], unawareness of illness [8], severe paralysis [8], spasticity [8], urinary and bowel incontinence [6, 8, 10, 12], limited postural control [5], hemianopsia [8], and dependence in activities of daily living [6–11]. Furthermore, in order to prevent missing potential relevant predictors of home discharge, we also performed a quick scan of studies performed among the general population of stroke patients for additional factors related to home discharge after stroke rehabilitation [13–22].

Based on these two groups of studies, five categories of factors measured at admission to rehabilitation are found to be negatively correlated to home discharge after rehabilitation of stroke patients:

1. *Demographic characteristics:* high age [5, 6, 13, 14, 16, 17, 19, 20, 22], non-white race [13], female sex [7, 13, 14, 17].
2. *Social and environmental characteristics:* living alone (i.e. not sharing a household) [7–10, 13–15, 17, 18, 21], absence of social support [7, 9–11, 18, 19], insufficient professional care [19], high need for home adaptations [19], and limited private financial means [19].
3. *Stroke related health status:* stroke history [13, 17], hemorrhagic stroke [ 7, 13, 17], more severe stroke [2, 16, 19, 22], larger stroke volume [13, 14, 16], loss of consciousness [8, 13, 16, 17, 19], cognitive disability [6–10, 12–17, 19], neglect [5, 7, 8, 14, 16, 17, 19], apraxia [16, 17, 19], unawareness of illness [8, 14, 17], severe paralysis [8, 14, 16, 17, 19], impairment in movement [17, 19, 20] spasticity [8], disorientation in time and place [16, 17, 19], emotional problems [13, 19], dysphagia [15, 16], urinary and bowel incontinence [6, 8, 10, 12, 13, 15–17, 19], limited postural control [5], restrictions in sitting balance [16, 19], and hemianopsia [8, 16, 17].
4. *General health status:* high blood pressure [13, 16], diabetes mellitus [13], pneumonia [13], cardiovascular disorders [13, 16], multimorbidity [13, 16], personality disorder [19].
5. *Functional status:* communication disability [19], low daily activity level [13], dependence in activities of daily living [6–13, 16, 17, 19–21].

The factors that were found to be related to home discharge in at least five of our selected studies were dependence in activities of daily living (*n* = 13 studies), cognitive disability (*n* = 12), living alone (*n* = 10), high age (*n* = 9), urinary and bowel incontinence (*n* = 9), neglect (*n* = 7), absence of social support (*n* = 6), loss of consciousness (*n* = 5), and severe paralysis (*n* = 5). Due to the large number of (potential) predictors of home discharge reported in literature, it is important for care professionals in intermediate care facilities for geriatric rehabilitation to gain insight in which factors most strongly correlate with home discharge among frail and multimorbid older stroke patients.

Therefore, the aim of this study is to identify which factors are associated with home discharge after inpatient rehabilitation among frail and multimorbid older stroke patients. For this purpose, in our study we have combined a set of factors previously found to be related to home discharge, in order to gain insight in the factors most strongly correlating with home discharge of frail and multimorbid stroke patients after inpatient geriatric rehabilitation.

## Methods

### Design

We performed a longitudinal cohort study, based on data from the MAESTRO-study [23] which is a two group multicenter randomized controlled trial evaluating the effects of a new geriatric rehabilitation program for older people with stroke admitted to an intermediate care facilities for geriatric rehabilitation. For this secondary analysis we used data of the patients allocated to the control group, who received usual care based on the Dutch guidelines for stroke rehabilitation [24]. Patients from the experimental group were excluded because of the possible intervention effect.

### Study sample

The sample for this study consisted of 92 persons admitted to an intermediate care facility for geriatric rehabilitation in the period November 2010 to December 2014. Inclusion criteria for these patients were: (1) age 65 year or older, (2) living independently in the community before stroke, and (3) being admitted to one of eight intermediate care facilities for geriatric rehabilitation in the south of the Netherlands under the prognosis that they would be able to return to their previous living situation after rehabilitation (as assessed 2 weeks after admission by clinical judgement of a multidisciplinary team at the intermediate care facility for geriatric rehabilitation). Patients, who were medically unstable or had severe cognitive disabilities and were unable to start rehabilitation, were excluded^23.^ Informed consent was obtained from all participants. The study protocol has been approved by the medical ethics committee of Maastricht University Medical Centre (MUMC+), the Netherlands (ISRCTN62286281, NTR2412). The study protocol has been published elsewhere [23].

### Data collection

Data were gathered by means of registration forms administered by care professionals of the intermediate care facility for geriatric rehabilitation and structured interviews with patients [23]. The interviews with the patients were conducted by trained research assistants at the start of the rehabilitation treatment.

#### Factors measured at admission to the intermediate care facility for geriatric rehabilitation

All potential predictors of home discharge of stroke patients after rehabilitation (described above) that were also measured in the MAESTRO study were selected for the present study. The final set of potentially predictive factors was divided in the five categories mentioned before: demographic characteristics, social and environmental factors, stroke related health status, general health status and functional status as presented below. The following 16 factors assessed at admission to the intermediate car facility for geriatric rehabilitation were available in the MAESTRO-dataset:

1. *Demographic characteristics*: age, sex;
2. *Social characteristics*: household situation before admission (living alone or with others);
3. *Stroke related health status*: stroke history, cognitive disability, neglect, apraxia, dysphagia, urinary and bowel incontinence, and sitting balance;
4. *General health status*: emotional problems, cardiovascular disorders, diabetes mellitus, multimorbidity;
5. *Functional status*: daily activity level, independence in activities of daily living.

Stroke history, neglect, apraxia, urinary and bowel incontinence, sitting balance, cardiovascular disorders and diabetes mellitus, were retrieved from patient records and dichotomized (present or not present). Information regarding household situation before admission (i.e. living alone or sharing a household with one or more persons) was assessed by means of the interview with the patient at admission to geriatric rehabilitation. In the same interview, also the factors emotional problems, multimorbidity, daily activity level, independence in activities of daily living and cognitive disability were assessed. Emotional problems were measured by the emotional problems domain of the EuroQol-5D (EQ-5D) [25]. This item was dichotomized in (0) no emotional problems, and (1) emotional problems. Multimorbidity was measured by a variable which included 17 different medical conditions which are scored as present (1) or not present (0) [26]. The summed multimorbidity score can range from 0 to 17 with higher scores indicating more conditions present. Daily activity level was measured by the Frenchay Activity Index (FAI) [27]. The FAI measures the daily activity level of stroke patients and consists of 15 items (range 15–60 with higher scores indicating better functioning)*.* The level of independence in activities of daily living was assessed with the Katz Index of Independence in Activities of Daily Living scale (Katz-15) [28] consisting of 15 items (range 0–15 with lower scores indicating a higher level of independence). Cognitive status was measured by the 11-item Minimal Mental State Examination (MMSE; range 0–30 with higher scores indicating better functioning) [29].

#### Discharge destination

Data regarding the living situation 6 months after admission (moment of discharge) to geriatric rehabilitation were gathered from the discharge registration of the eight participating rehabilitation units. The available data were dichotomized into (1) discharged to the previous living situation (i.e. home discharge) and (0) not discharged to the previous living situation (i.e. still in geriatric rehabilitation or admitted to nursing home, care home or service apartment).

### Statistical analysis

First, descriptive statistics were used to calculate means or proportions of the potential prognostic factors. Second, a Pearson R correlation analysis was applied to assess strength of the univariate relationship between the potential prognostic factors, and discharge destination. For some categorical factors (i.e. gender, household situation, apraxia, neglect, dysphagia) a chi-square test was applied. Pearson correlation is a measure of strength, whereas Ch-square is a test statistics. All categorical variables are dichotomous. Thus a Pearson correlation can be calculated (instead of phi coefficient; they are exactly the same). Third, a two-level logistic regression analysis was conducted to study the relationship between the potential prognostic factors and discharge destination. The first level consists of the patients and the second level consists of the organizations, because the patients are nested within the organizations. In each step of the analysis the factor with the highest *p*-value was eliminated until only factors remained with a p-value below 0.10. The association of each individual variable was expressed in an odds ratio, 95% confidence interval, and p-value. All statistical analyses were conducted using SPSS software version 25 for Windows.

## Results

Patient characteristics measured at baseline are presented in Table 1. The mean age of the patients was 79.0 (SD 6.4) year with a range of 65 to 94 years. About half of the patients (*n* = 47, 51.1%) were female and 43 patients (47.3%) lived alone before admission. On average, the patients had four different medical conditions. After 6 months 71 patients (77.1%) had returned to their former living situation, and 21 (22.8%) patients were admitted to sheltered housing or nursing home see Table 2).

**Table 1:** Patient characteristics measured at baseline (*n* = 92)

Demographic characteristics	Scores
Mean age (SD)	79.0 (6.4)
Female gender N (%)	47 (51.1)
Social characteristics	
Household situation: living alone N (%)	43 (47.3)
Stroke related health status	
Stroke history N (%)	28 (29.2)
Cognitive disability (MMSE) Mean (range)	22 (0–30)^a^
Neglect N (%)	21 (21.9)
Apraxia N (%)	23 (24.0)
Dysphagia N (%)	28 (30.8)
Urinary and bowel incontinence N (%)	34 (37.0)
Restrictions in sitting balance N (%)	73 (76.0)
General health status	
Emotional problems N (%)	38 (41.3)
Cardiovascular disorders N (%)	22 (23.9)
Diabetes mellitus N (%)	25 (27.2)
Multimorbidity Mean (SD)	4 (1.67)
Functional status	
Daily activity level (FAI) Mean (range)	38(15–45)^a^
Independence in activities of daily living (Katz-15) Mean (range)	6 (0–15)^a^

^a^the underlined score is the most favorable score

**Table 2:** Discharge destination of the patients after 6 months

Discharge destination	*n* = 92
Discharged to former living situation N (%)	71 (77.1)
Discharged to other setting N (%)	21 (22.8)
• Sheltered housing N (%)	1 (1.1)
• Care home (%)	13 (14.1)
• Nursing home N (%)	7 (7.6)

Table 3 presents the bivariate correlations between the 16 included prognostic factors and discharge destination. The analysis shows that only one of the 16 potential prognostic factors, independence in activities of daily living, is significantly related to home discharge (*r* = − 0.38, *p* = 0.00). The logistic regression analysis presented in Table 4 also shows that only a higher level of independence in activities of daily living is significantly related to home discharge (OR = 0.70, *p* = 0.01).

**Table 3:** Bivariate Correlation analyses of predictive factors and discharge to former living situation

Predictive factor	Pearson r	*P*
Demographic characteristics		
Age	−0.04	0.69
Gender	−0.09	0.37
Social characteristics		
Household situation: living alone	0.14	0.20
Stroke related health status		
Stroke history	0.12	0.26
Cognitive disability (MMSE)	0.09	0.38
Neglect	− 0.03	0.80
Apraxia	− 0.06	0.56
Dysphagia	− 0.13	0.23
Urinary and bowel incontinence	0.08	0.47
Restrictions in sitting balance	0.12	0.25
General health status		
Emotional problems	− 0.15	0.16
Cardiovascular disorders	− 0.01	0.90
Diabetes mellitus	0.09	0.42
Multimorbidity	−0.10	0.35
Functional status		
Daily activity level (FAI)	0.01	0.96
Independence in activities of daily living (Katz-15)	− 0.38	0.00*

*=significant at 0.05 level

**Table 4:** Logistic regression analyses of associated home discharge predictors

Predictive factors	OR	*P*	95% CI for OR
Demographic Characteristics			
Age	0.97	0.68	0.84–1.12
Gender	1.93	0.48	0.31–11.89
Social characteristics			
Household situation: living alone	1.95	0.42	0.37–10.26
Stroke related health status			
Stroke history	2.72	0.26	0.46–15.95
Cognitive disability (MMSE)	0.99	0.88	0.83–1.17
Neglect	0.66	0.62	0.13–3.47
Apraxia	1.01	1.00	0.15–6.58
Dysphagia	1.33	0.73	0.27–6.61
Urinary and bowel incontinence	1.25	0.79	0.23–6.68
Restrictions in sitting balance	1.14	0.87	0.22–6.05
General health status			
Emotional problems	0.48	0.34	0.11–2.17
Cardiovascular disorders	0.93	0.94	0.17–5.24
Diabetes mellitus	3.86	0.18	0.53–28.28
Multimorbidity	0.83	0.46	0.49–1.39
Functional status			
Daily activity level (FAI)	1.02	0.78	0.90–1.15
Independence in activities of daily living (Katz-15)	0.70	0.01*	0.53–0.93

*significant at 0.05 level, ICC of the two-level model is 0.32

## Discussion

In the Netherlands, specialized intermediate care facilities for geriatric rehabilitation aim to enable community-living frail older stroke patients to return to their previous living situation after rehabilitation. However, due to the complex nature of stroke, and the frailty level of these older multimorbid stroke patients (as indicated by the average number of four medical conditions), predicting functional recovery and discharge destination are considered very challenging.

In the present study, we examined 16 factors that, based on the literature, might be potentially associated with discharge destination of older stroke patients admitted to geriatric rehabilitation. These potential prognostic factors were: age; sex; household situation before admission; stroke history; cognitive disability; neglect; apraxia; dysphagia; urinary and bowel incontinence; emotional problems; cardiovascular disorders; diabetes mellitus; multimorbidity; sitting balance; daily activity level; and independence in activities of daily living. A two-level multivariable logistic regression analysis revealed that only a higher level of independence in activities of daily living at admission (as measured with Katz-15) was significantly associated with being discharged to the former living situation within 6 months after admission to geriatric rehabilitation. The fifteen other factors were not significantly associated with home discharge.

Our results regarding the relationship between level of independence in activities of daily living at admission and discharge destination after rehabilitation are in accordance with results of previous studies in the general population of stroke patients [13, 16, 17, 19–21] and among older stroke patients [6–12], which showed that independence in activities of daily living was the most frequently mentioned predictor in the studies included in our literature search.

However, for the other fifteen prognostic factors, no significant association with discharge destination in our sample of frail and multimorbid older stroke patients could be identified. This is rather unexpected, because a significant relationship of these prognostic factors with discharge destination was observed in one or more previous studies among the general and/or older population of stroke patients [5–10, 12–22].. The fact that our findings are inconsistent with current literature can be explained by several factors. First, we also included prognostic factors in our analysis that were only reported in studies among the general population of stroke patients (i.e. apraxia, dysphagia, sitting balance, emotional problems, cardiovascular disease, diabetes mellitus, and daily activity level). It is likely that our sample of geriatric rehabilitation patients is considerably more complex compared to the general population of stroke patients because geriatric rehabilitation patients are often frail, multimorbid and may also have a weaker social network, so there might be other prognostic factors present which can potentially influence the chances of home discharge. However, the majority of prognostic factors included in our analyses were (also) reported by studies among the population of older stroke patients who received rehabilitation in an intermediate care facility. A second possible explanation is that there are considerable differences between our study sample and the samples of the majority of these other studies. Our study sample consisted of frail and multimorbid stroke patients, and it is unclear whether studies performed in other countries included a comparable frail and multimorbid population. In addition, in the Netherlands people with severe cognitive impairments (such as dementia) are in general not admitted to geriatric rehabilitation due to a lack of trainability. It is possible that in countries where persons with severe cognitive impairments can be admitted to geriatric rehabilitation, cognitive impairment might be a statistically significant predictor of home discharge.

A third explanation might be the fact that some of the prognostic factors included in our study, are measured in a different way compared to previous studies. Instruments can differ for example with regard to their sensitivity or with regard to the specific aspects of the same phenomenon they assess, which might have resulted in different correlations.

This study has several limitations. First, several prognostic factors were measured in a dichotomous way, such as sitting balance, apraxia and neglect, which may have resulted in some loss of information. It is possible that a more comprehensive way of assessing these factors would have led to other results in our analysis. Second, this study is a secondary analysis of existing data. For this reason, we were not able to include all potential relevant predictors of home discharge in our study found in previous studies among older patients admitted to intermediate care facilities for rehabilitation, including social support [7, 9–11], hemorrhagic stroke [ 7], loss of consciousness [8], unawareness of illness [8], severe paralysis [8], spasticity [8], postural control [5], and hemianopsia [6–11]. Most of these factors were only found in one single or a few studies, however social support was found in six other studies, and loss of consciousness and severe paralysis in five studies, so it remains unclear whether these factors might also be relevant predictors in our frail population. Although household situation (i.e. living alone versus living with others) might be considered an indicator of social support it seems likely that this variable does not differentiate enough within our frail population.

Almost half (47%) of our population lives alone, and probably a considerable number of the other half has a partner who is also frail and needs support. Therefore, in a frail and multimorbid population, it might be better to assess the availability of informal caregivers, and social support in a more comprehensive way. Therefore, it is possible that we missed some relevant prognostic factors especially in the domain of social support. Furthermore, researchers in the domain of stroke rehabilitation in frail older people might have collectively missed or understudied potential relevant prognostic factors for home discharge, such as the level of frailty, (post stroke) depression, availability of family caregivers and/or professional caregivers, motivation and preferences of patients and family caregivers, and financial means. A third limitation is the size of our sample. Although bivariate analyses revealed that only a higher level of independence in activities of daily living at admission was significantly related to home discharge, for the logistic regression analyses our sample size can be considered relatively small in relation to the relatively large number of prognostic factors in our logistic regression. However, bivariate analysis also revealed no significant correlations between the other prognostic factors and discharge destination. A fourth limitation is the fact that our study is performed in only one country (the Netherlands). It is possible that due to cultural differences and/or differences in healthcare systems, in other countries different factors might be relevant for home discharge after stroke rehabilitation among frail older persons.

## Conclusion

In conclusion, our study shows that the vast majority of prognostic factors reported in literature to be related to home discharge among stroke patients after rehabilitation, were not correlated to home discharge within our study sample of frail and multimorbid older persons admitted to geriatric rehabilitation. Our analyses showed that only a higher level of independence in activities of daily living at admission to geriatric rehabilitation is associated with discharge to the former living situation, 6 months after starting stroke rehabilitation. It is important to gain additional insight in possible other factors that might predict home discharge among frail older stroke patients after geriatric rehabilitation, such as the level of frailty, factors related to social support, the availability of family and/or caregivers, and motivational factors.

## Data Availability

The datasets used and/or analysed during the current study are available from the corresponding author on reasonable request.
